# The Virtual Skeleton Database: An Open Access Repository for Biomedical Research and Collaboration

**DOI:** 10.2196/jmir.2930

**Published:** 2013-11-12

**Authors:** Michael Kistler, Serena Bonaretti, Marcel Pfahrer, Roman Niklaus, Philippe Büchler

**Affiliations:** ^1^Institute for Surgical Technology and BiomechanicsUniversity of BernBernSwitzerland; ^2^UCSF School of MedicineRadiology and Biomedical ImagingUniversity of CaliforniaSan Francisco, CAUnited States; ^3^Engineering and Information TechnologyBern University of Applied SciencesBielSwitzerland

**Keywords:** medical informatics, Internet, image processing, computer-assisted, demographic analysis, statistical models

## Abstract

**Background:**

Statistical shape models are widely used in biomedical research. They are routinely implemented for automatic image segmentation or object identification in medical images. In these fields, however, the acquisition of the large training datasets, required to develop these models, is usually a time-consuming process. Even after this effort, the collections of datasets are often lost or mishandled resulting in replication of work.

**Objective:**

To solve these problems, the Virtual Skeleton Database (VSD) is proposed as a centralized storage system where the data necessary to build statistical shape models can be stored and shared.

**Methods:**

The VSD provides an online repository system tailored to the needs of the medical research community. The processing of the most common image file types, a statistical shape model framework, and an ontology-based search provide the generic tools to store, exchange, and retrieve digital medical datasets. The hosted data are accessible to the community, and collaborative research catalyzes their productivity.

**Results:**

To illustrate the need for an online repository for medical research, three exemplary projects of the VSD are presented: (1) an international collaboration to achieve improvement in cochlear surgery and implant optimization, (2) a population-based analysis of femoral fracture risk between genders, and (3) an online application developed for the evaluation and comparison of the segmentation of brain tumors.

**Conclusions:**

The VSD is a novel system for scientific collaboration for the medical image community with a data-centric concept and semantically driven search option for anatomical structures. The repository has been proven to be a useful tool for collaborative model building, as a resource for biomechanical population studies, or to enhance segmentation algorithms.

## Introduction

Beyond the obvious variability in appearance, a large anatomical variation among the human population exists. For example, even for similar looking people, the human skeleton has a large variability in bone shape, internal structure, and mechanical strength. Following the systematic use of modern imaging techniques in the medical routine, the interest in studying human variability grew in the medical research community. Roughly two decades ago, Cootes et al [1] introduced the first statistical model to quantify anatomical shapes [2]. This technique became very popular in the medical image analysis community and has been used in a wide range of applications: novel implant design [3,4], surgical planning [5,6], and improving image segmentation [7,8] and image registration [9]. More recently, these models have been extended to combine the description of the anatomical shape with mechanical information to analyze the risk of fracture in a population [10,11] or to design orthopedic implants [12].

A recurrent problem that is faced when applying statistical shape model (SSM) to specific clinical indications is the large amount of data required to build a valid model. A repository for images and processed data is therefore vital for the fast development of new applications or image processing algorithms based on this technology. However, the existing solutions for storing medical images are not appropriate for building statistical models. In the clinical environment, picture archiving and communication system (PACS) was introduced in the 1980s to manage clinical images of patients [13,14]. The digital imaging and communications in medicine (DICOM) is the standard file format used in PACS, which ensures that the proper metadata concerning the patient and the image technique are available to the radiologist. This system is ideal for archiving clinical data but is ineffective if the user wants to retrieve a large amount of data corresponding to the same anatomical site. In addition, due to legal restrictions and data protection laws, it is not possible for researchers to freely access the large database of medical imagery stored in clinical institutions. Therefore, significant efforts are necessary to obtain data, to process them, and to build the models. However, even after this time-consuming process, collections of data are often lost or mishandled in research institutions, resulting in replication of work, even within the same institution. A centrally organized system can limit or even suppress these barriers, which hinders fast and innovative approaches in medical image research.

Inspired by PACS and institutional repository software [15-17], several projects have been developed for the biomedical research community. For example, the Extensible Neuroimaging Archive Toolkit (XNAT) [18] has developed an open source solution for the neuroimaging research community. Recently, the Midas media archiving platform [19] was developed to extend the functionality of DSpace [15] repository software. This platform aims to simplify the collaboration between researchers and to provide more flexibility on the supported file format and data structure. Several other projects are building infrastructures to host data collections in their respective research domain. For example, the Living Human Digital Library [20] aims to provide a collection of raw and processed data on the anatomo-functional characteristics of the human musculoskeletal system at dimensional scales spanning from the whole body down to the molecules. However, this system is limited to a small number of human bodies generated during the project and does not allow the user to upload their own datasets. The Internet Brain Segmentation Repository [21] provides manually guided expert segmentation along with magnetic resonance brain image data. All these projects provide extremely important data to the scientific community, but they either consider a limited number of subjects, are specific to a certain organ or pathology, or present a mix of patients and pathology. A general database where medical image datasets are stored and further processed toward statistical shape modeling is required to model the variability of the complete human anatomy.

The objective of the Virtual Skeleton Database (VSD) is to provide and maintain an open access repository for scientific researchers to archive, search, preserve, trace, and share data resources for statistical models. In this paper, the authors describe the main concepts implemented in the system and demonstrate its operation on exemplary use cases.

## Methods

### General Concepts

The VSD can be described as a catalog of data that is accessible and organized with a focus on, but not limited to, building statistical anatomical models. Therefore, the VSD was built around the concept of “data objects”, which constitute the basic element of the system. These data objects can be any type of image file, processed data, or models. This approach provides a large flexibility to the system in terms of data formats, data organization, and data collaboration. The design is intended to have the capacity to provide the same basic functionality as a PACS, but it is more flexible and can integrate additional research data, such as labeled images and statistical models.

### Technology

**Figure 1:**
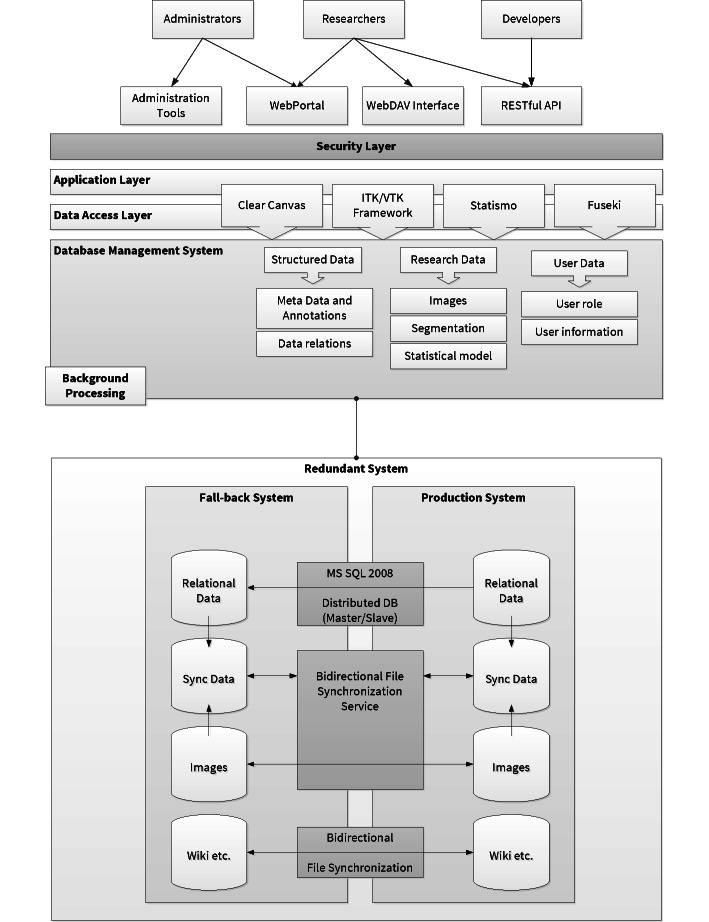
Schematics of the multitier architecture of the VSD, which consists of different types of interfaces, an application logic and security layer, and data access and data storage.

The architecture of the VSD is based on the classical multitier architecture (see Figure 1). The topmost layer provides multiple interfaces: the Web portal for managing and sharing the data, Web-based Distributed Authoring and Versioning protocol (WebDAV) for direct interaction with different file systems, and a Representational State Transfer interface (REST), an application programming interface for interaction with other scientific applications while the administration functionality is provided through a Web application. The middle tier provides all the necessary application logic but also contains the functionality covering security aspects. Data storage and data access are provided on the next tier. Aside from the relational database system for storing the information, the system integrates other tools and frameworks for specific purposes; ClearCanvas is used to handle DICOM files, Insight Toolkit, Visual Toolkit (ITK/VTK) for image processing, Statismo for statistical shape models, and Fuseki for the integration of ontologies. The scalable data layer (database and data) is equipped with a mechanism to provide a full synchronization between two functional redundant systems.

The development of the VSD was based mainly on Microsoft technologies. The system relies on a Microsoft Structured Query Language (SQL) database and the Microsoft Internet Information Service (IIS) Web-server. For the application development, Microsoft Razor, which is built on the Microsoft .NET technology, was chosen to enable consistent development of a responsive Web service. The Razor application framework was complemented by the established frameworks Bootstrap [22] and jQuery [23] for a smooth and dynamic user experience on the website (see Multimedia Appendix 1).

### Registration

**Figure 2:**
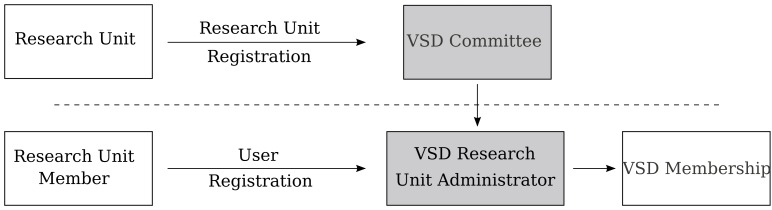
Registration process - Top: registration of a new research unit and appointment of a research unit administrator. Bottom: individual users can register to a research unit and have to be accepted by the respective administrator.

Standard user and object rights management is required to control access, visibility, and sharing of the objects stored on the VSD. However, since legal and ethical aspects play an important role when medical images are involved, a two-step registration procedure and user management were implemented. The system’s registration procedure was designed to build the VSD as a research-only repository (see Figure 2). In the first step, academic institutions can register as research units. After approval of the research unit by the VSD administrator, each research unit can administer their members and is responsible for controlling access to the research data and ensuring the proper rights and licenses are attached to their uploaded datasets.

### Medical Images

**Figure 3:**
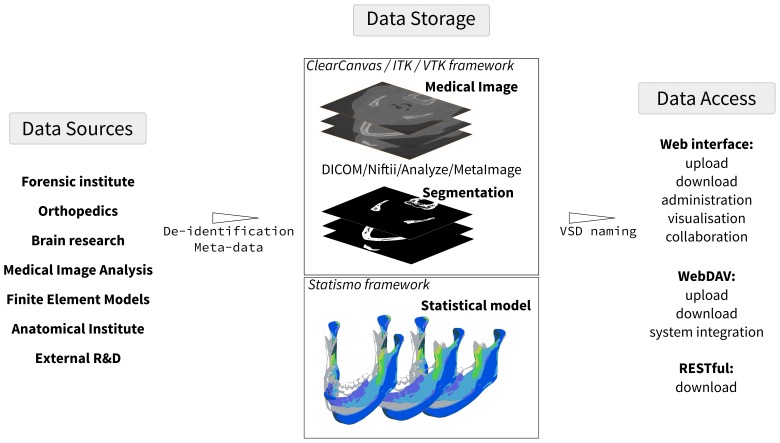
Conceptual overview of the VSD - data from various sources are stored in the VSD and can be accessed, organized, and downloaded.

The system provides the user with multiple options for the image upload. A standard Web upload form and a Java applet are available. While the standard browser upload is most convenient for small images, the Java applet is able to submit files larger than 2GB to the server. Additionally, if close integration in the local system is required, the VSD can be accessed through the WebDAV, which allows for customized upload workflows. The VSD integrates the most common image formats used by the medical image analysis community, but theoretically any kind of medical image format could be integrated. The VSD can read classical DICOM files through the ClearCanvas framework, and image types recognized by the ITK library (MetaImage, Niftii, and Analyze) are accepted (see Figure 3). During the upload process, metadata are extracted from the images, preview images are generated, and a de-identification of the medical image is performed. The de-identification consists of file type-specific header manipulation and the replacement of the original file name by a generic VSD file name, preserving the information only for the original contributor. To keep the storage system efficient, the uploading functionality has mechanisms to avoid duplicated objects on the disk and supports multiple versions of the objects.

To assign proper and consistent metadata to the images, the VSD features a publishing concept where uploaded data have to be reviewed before they become available to the other users. During this process, the user is asked to review the uploaded dataset and to provide the missing information. First, the dataset has to be characterized by annotating the anatomical structure depicted in the image. For this metadata, the anatomical terms are taken from the comprehensive Foundational Model of Anatomy (FMA) ontology [24]. This ontology includes more than 75,000 anatomical terms and 450,000 direct relations between classes. Additional metadata like age, gender, and image modality can be filled to enrich the meta-information content of the image. If a related dataset already exists on the VSD, the user can reference this data by linking both datasets together. For example, different image modalities of the same patient and anatomical structure can be linked and therefore grant a direct access between both datasets. As a last step, a Creative Commons license can be attached to a dataset to specify the intended offline usage. To control the sharing of the data on the website, predefined permission sets are available to specify the access rights. After a final activation, the data are accessible to other users of the VSD.

### Data Organization

**Figure 4:**
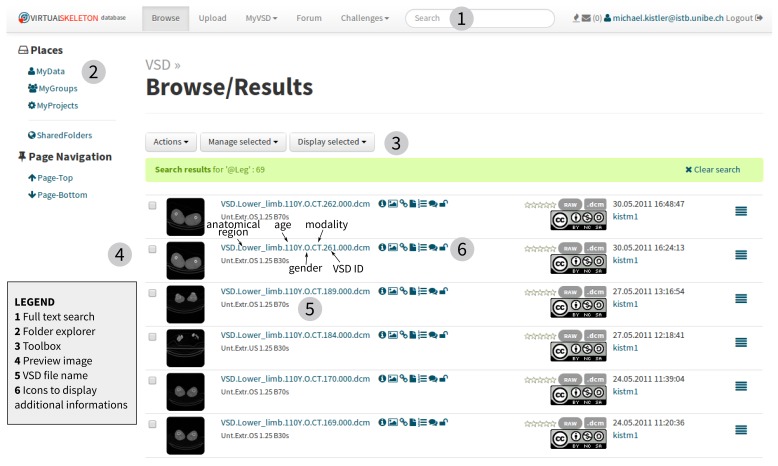
VSD Web interface to browse data with folder explorer: MyData=Location of the user’s uploaded data; MyGroups=Default collaboration folder accessible to all group members; MyProjects=Folders to organize data into personal projects; SharedFolders=Folders of other VSD members which are shared to the user.

Users can search the available online data catalog to find the datasets relevant for their projects (see Figure 4 and Multimedia Appendices 2 and 3). The system allows the creation of virtual folders to organize the available datasets, which provides the user with the flexibility to freely create their personal workspace. Data objects are not physically moved or duplicated, but the system creates a reference to the data object, retaining the original file permission and ownership. For collaboration or other purposes, the user can share parts of their workspace by changing the permission of the folder accordingly. To simplify the collaboration within a research unit, the system provides a default shared group folder, which is accessible and manageable by all members of the research unit.

Several functions provide additional information to the users and help them identify the relevant datasets. Each dataset can be rated for quality and tagged with personal comments. To comment on the object publicly, a discussion thread can be initiated in the integrated VSD forum (see Multimedia Appendices 4 and 5). Additionally, the search function allows users to find specific metadata including ontology terms. The ontology search is using SPARQL Protocol and RDF Query Language (SPARQL), which queries the semantic information contained in the ontology. This feature enables a powerful search where anatomical substructures can be identified within stored data objects. For example, a user can search for images containing the body part “Leg”. With a conventional index-based search, only data annotated as “Leg” can be retrieved (zero search results in the VSD). With the semantics of the ontology, the VSD will find and return data that are or contain the Leg (69 datasets found in the VSD).

### Retrieve Images

An individual dataset as well as a folder structure can be downloaded from the VSD for processing on a local machine. The data are wrapped for download into a compressed archive file to reduce file size and to preserve the folder structure from the VSD. For local access to folders on the VSD, the user can mount the WebDAV folder as a network drive on their computer.

### Segmented Images

Images that contain segmentation labels can be uploaded the same way as medical images. The system identifies how many anatomical structures are labeled in the segmentation image, the de-identification is performed, and the thumbnail images for preview are extracted. During the review process, all the labels of anatomical structures have to be specified and the segmentation technique can be described. The uploaded segmentation data can then be linked to original medical images or to other segmentations of the same structure, which have been performed using a different technique.

### Statistical Models

To standardize the building and storage of shape models, the open source library “Statismo” has been developed [25]. An important aspect of this library is that it proposes a simple file format to store shape model, based on the Hierarchical Data Format (HDF5). The VSD recognizes shape models stored in HDF5 and is able to extract important information about the model, such as the datasets used or the registration technique used. After the upload, the review process is similar to that of the images, asking the user to annotate the anatomical region and to specify the license and access rights.

Three exemplary projects using key functionalities of the VSD are presented. First, the creation of a statistical model of the cochlea for surgery and implant optimization is described. As a second example, a statistical appearance model of the femur is used to analyze fracture risk within a population. Finally, an online segmentation challenge hosted by the VSD is presented.

## Results

### International Collaboration on Statistical Shape Model of the Cochlea

**Figure 5:**
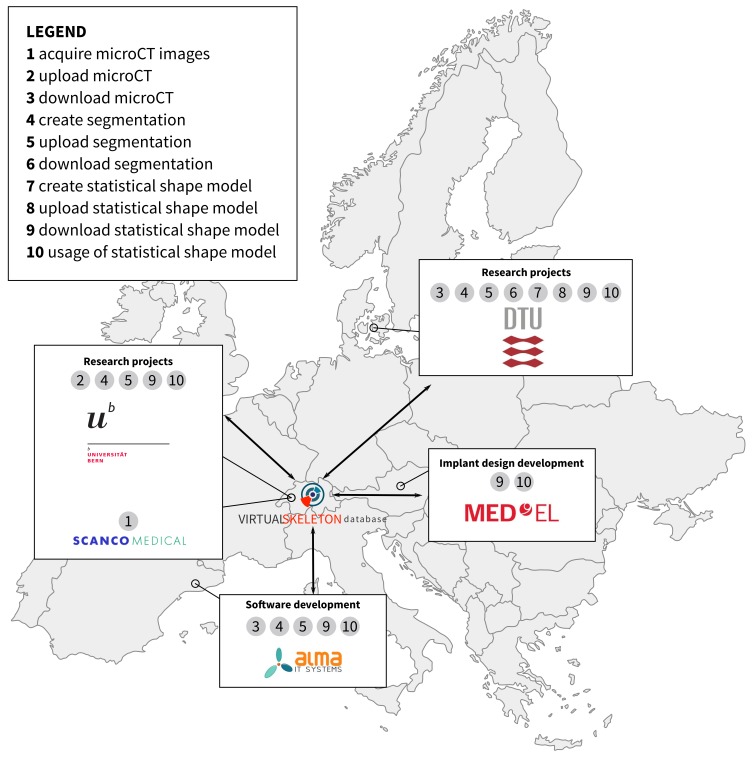
Collaborative network, interactions, and workflows for the Hear-eu project on cochlear implants.

The goal of this international collaboration project, the Hear-EU project [26], is to improve cochlear implants. This surgical procedure tries to overcome hearing loss by implanting a device that accesses the cochlea of the inner ear. Clinical expertise and detailed information about critical structures close to the surgical site are needed for such a surgery. Since this surgical intervention is very complex and a vast variety of patient anatomies exist, the project hypothesizes that a comprehensive knowledge of the involved anatomical structures would enable improvements for implant design and for surgical planning. In this context, the VSD acts as a data hub (see Figure 5; map adapted from [27]) to assist the building of a statistical shape model of both the middle and the inner ear. High resolution computed tomography (CT) images with a data size of over 5GB are uploaded to the VSD and will be processed by different institutions across Europe. The resulting segmentation images will be subsequently re-uploaded to the VSD. Having both the images and processed data available will enable the Technical University of Denmark to create a statistical shape model of the regions of interest. Finally, the shape models will be available on the VSD and can be used by different teams to optimize surgical procedure as well as the functional outcome of the surgery on a patient-specific basis.

###  Biomechanical Femur Population Study

The authors searched the VSD for available lower extremities datasets to conduct a study on the fracture risk on this population. A total of 72 female and 72 male subjects were isolated and retrieved together with their available metadata. The population consisted mainly of Caucasian (114) supplemented with Asian (28) and African (2) subjects. The female subpopulation’s body mass index (mean 26.4, SD 5.5) was slightly higher compared to the male (mean 25.5, SD 4.9) subjects, while the male represented an older subpopulation than the female group.

For each gender, the statistical appearance model was calculated using the image-based approach described by Bonaretti et al [28] and polyaffine demons [29]. From both appearance models, 1000 instances were created and biomechanical finite element simulations were performed with a loading situation corresponding to normal walking [30,31]. The bone intensity values were converted into material properties [32,33] and mapped onto each node of the FE (finite element) mesh of the second order tetrahedral elements.

**Figure 6:**
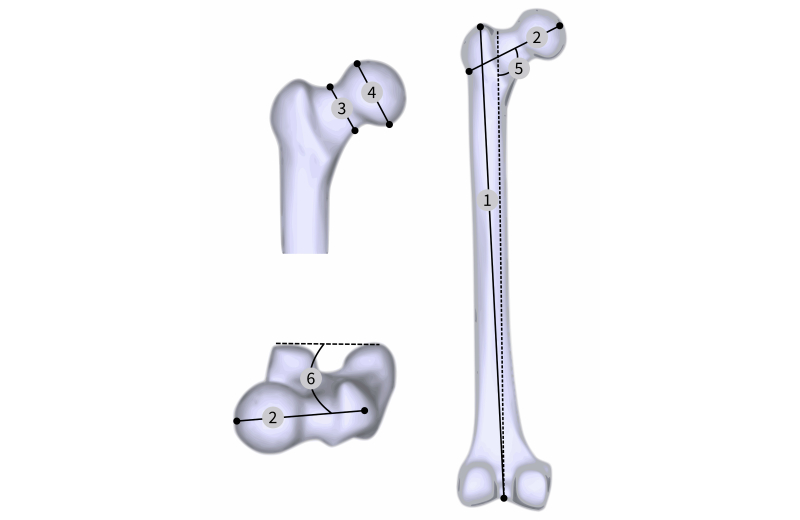
Geometrical measures: (1) femur length, (2) neck length, (3) neck width, (4) femoral head width, (5) neck-shaft angle, (6) anteversion angle.

The relationships between the fracture risk in the femoral neck and morphometric measurement was performed (see Table 1). Six geometrical measures were identified [34,35] (see Figure 6) as possible risk predictors. As a mechanical parameter, the average Young’s modulus in the femoral neck was calculated. All predictor parameters were centered and normalized to perform the regression and evaluate their influence in femur fracture risk.

For both genders, body weight was identified as the most relevant predictor for fracture risk while the mechanical measures showed a lower influence of fracture risk in general. In contrast, among geometrical parameters, the neck-shaft angle could be identified as playing a predominant role for both populations, whereas the order of importance of the parameters shows clear difference between men and women. The complete study results are presented by Bonaretti [28].

### Segmentation Challenge for Brain Tumors

**Figure 7:**
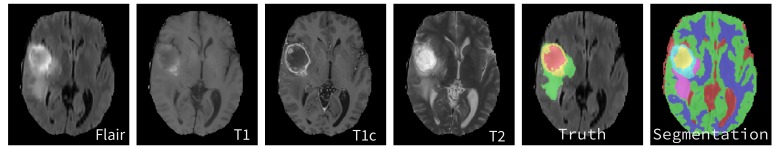
Exemplary multimodal MR images of a high-grade Glioma patient 0001 with truth and label result from an automatic segmentation of a BRATS participant (red=cranio spinal fluid, green=gray matter, blue=white matter, yellow=necrotic, turquoise=active tumor, pink=edema).

The VSD was chosen by the Multimodal Brain Tumor Segmentation (BRATS) challenge organizer to host their data and challenge. The data were publicly available to any team around the world to develop and train their segmentation algorithms. In addition, the VSD was used to evaluate the submission of the competitor during the challenge, which took place during the annual conference of the Medical Image Computing and Computer Assisted Intervention (MICCAI) Conference 2012. Hosting such a challenge required several functionalities, which illustrates most of the features of the system. The VSD hosted multicenter magnetic resonance (MR) imaging sets and their related ground truth, which were defined by inter-reader agreement from clinicians. The clinical segmentations were used to determine the accuracy of the segmentation of tumor subregions. A total of 80 brain MR and segmentations were available to the competitors for training purposes (see Figure 7). An additional 30 challenge datasets which consist only of MR data were available for download. Their respective ground truth segmentation was hosted on the VSD but the download was protected through appropriate file permission. The users uploaded their segmentation through the Web interface, reviewed the uploaded segmentation, and started an automatic evaluation process. The VSD automatically identified the ground truth corresponding to the uploaded segmentations. The evaluation of the different standard parameters used to evaluate the quality of the segmentation (such as Dice coefficients) runs in the background and takes less than 1 minute per segmentation. Individual and overall results of the evaluation were automatically published on the VSD webpage and were downloadable as a comma separated values (CSV) file for further statistical calculations (see Multimedia Appendices 6 and 7). The VSD has evaluated more than 3000 segmentations and had over 80 registered users for the BRATS 2012 group. The service was successfully used for the challenge in 2012 when around 700 evaluations were handled by the system within the last 2 hours of the competition. The results were presented in the proceedings of MICCAI 2012 [36], and the data are now open to anyone who wants to evaluate new segmentation tools against previous BRATS evaluation sets. So far, around 15,000 additional segmentations have been successfully benchmarked by the VSD.

**Table 1:** Measured bone geometry and bone mechanical properties for men and women.

Parameter	Male mean (SD)	Female mean (SD)
Femur length, cm	42.6 (2.9)	40.8 (2.5)
Neck length, cm	10.5 (0.8)	9.9 (0.5)
Neck width, cm	3.9 (0.3)	3.6 (0.2)
Head width, cm	5.0 (0.5)	4.5 (0.5)
Neck-shaft angle, degree	128 (4)	130 (4)
Anteversion angle, degree	15 (7)	19 (7)
Young’s modulus, GPa	2.7 (0.7)	3.4 (0.5)
Body weight, kg	64 (14)	60 (14)

## Discussion

### Principal Findings

The VSD features various innovative approaches to provide an efficient and easy-to-use open access repository to the medical image and modeling community focusing on the creation of statistical models. The standard image and segmentation file types (DICOM, MetaImage, Analyze, and Niftii) can be read and stored by the system. Additionally, the Statismo framework with a standard file type for statistical models has been included, which allows statistical models to be shared, hosted, and collaborated on via the VSD. The complete data used in the process of building a statistical shape model can be stored and tracked through the related data information in the VSD (see Multimedia Appendices 8 and 9 ). Built in as generic a way as possible, the VSD hosts a catalog of data objects, which can be individually organized by users according to their needs. The anatomical structures are annotated using a comprehensive ontology allowing the integrated search algorithm to identify anatomical structures within datasets, which is not possible with a standard index-based search.

Access to the VSD is open to research units and their members. Users are invited to contribute by uploading images and processed data, which will increase the data pool available on the VSD. By adding their data to the VSD, the users profit from the permanent accessibility provided through the Web service. This approach supports the current trend towards open access and re-usability of research results, which should be accompanied by the policy of scientific journals (see Multimedia Appendix 10). This facilitated access to data should enhance productivity and research quality through competition and stimulate improvements by the research community. The three example projects presented highlight the benefits of the VSD and its contribution to statistical model building, population-based research, and object identification in medical images.

### International Collaboration on Statistical Shape Model of the Cochlea

With the VSD, it is possible to streamline the process of statistical shape model building for accurate population-based models in a multidisciplinary project setting. All parties have access to the same dataset and the metadata. The individual processing tasks can be distributed while the research data is stored on the VSD. An institution that has access to high resolution medical data can provide researchers with medical image analysis background with data for segmentation. Once these segmentations are available on the VSD, the statistical shape model can be engineered and provided through the VSD. The statistical model then can be implemented into surgical planning, segmentation algorithms, or implant design improvements. Since every processing step is stored on the system, this approach ensures a high level of reproducibility of the model building and quality control.

### Biomechanical Femur Population Study

The VSD provides a service to overcome the limitation of the small number of available subjects in mechanical studies by promoting statistical shape model, hosting the respective images and processed data. This approach enables population-based studies. These population-based investigations allow not only study of the mechanical behavior of a large amount of bones, but also the comparison among different groups of populations. They allow study of the shape and volumes of anatomical structures and, consequently, clinically relevant parameters. As presented, for geometrical parameters of the femur, the neck-shaft angle plays a predominant role for fracture risk of both genders. Additionally, differences between men and women could be identified due to the use of a population-based model. Similar approaches could be used to perform virtual evaluation of orthopedic implants and optimize their design to market-specific needs.

### Segmentation Challenge for Brain Tumors

Accurate segmentation is a critical step in visualization for treatment planning or later to monitor the therapeutic outcome. Manual segmentation from experienced experts is generally considered to contain the most reliable information. However, a lot of effort goes into the development of algorithms to automate this process. However, for almost every anatomical structure, those algorithms have to be adapted or trained. As a consequence, these techniques have to be constantly enhanced. To catalyze the innovation, researchers need image data and the opportunity to compare their results. The VSD provides the data repository and an evaluation platform, which are both needed to enhance those algorithms. The usefulness of such a platform in order to improve the sensitivity and specificity of the identification of glioblastoma is documented by the activity and registration numbers on the VSD. Due to the object-centric architecture of the system, the VSD is able to host any similar setup for other anatomical structures.

### Limitations and Further Directions

Although already populated with an initial set of data, the collection still needs to grow significantly. Constant efforts are being made to increase the amount of data, which are also expected to increase with the dissemination activity and number of users. Additionally, the VSD is expanding the supported file type based on requests from users. Finally, an additional interface to exchange data with the system is planned based on the REST interface, which will allow for online preparation of statistical models.

To deal with the delicate aspect of data privacy, the VSD provides automatic tools such as de-identification, metadata stripping, and file name replacement. However, these tools cannot prevent the possible identification of a patient associated with objects visible in the medical images themselves. In some case, a special plate or implant, a rare malformation, or the image of the head is sufficient to recognize the subject. Therefore, the owner of the data is responsible for ensuring that they have the right to share the images and that a proper anonymization has been performed by removing identifiable regions of the image. However, the functionality on the VSD to give the data a two-level control—permission and license—allows the user to ensure that images are accessible only to individuals allowed to work with their data.

### Conclusions

The VSD allows users to very flexibly manage and organize their data and online workspace. The VSD provides researchers with access to a growing community-filled data repository. The authors showed a novel system for scientific collaboration in the medical image community with a unique object-centric concept and semantically driven search option for anatomical structures for medical images, processed data, and statistical models. However, the clientele is not limited to these communities since the system can and will deliver functionality for other file types and their applications, for example, finite element models. We have presented three applications that benefit from such a Web application. The VSD is a valuable solution especially in the context of the growing needs of open access, re-usability, and reproducibility of scientific work.

## Multimedia Appendix 1

Front page of the Virtual Skeleton Database (VSD).

## Multimedia Appendix 2

List view of the full body dataset number 0001.

## Multimedia Appendix 3

Expanded view of the full body dataset showing additional information and previews.

## Multimedia Appendix 4

Forum start page.

## Multimedia Appendix 5

Forum thread of an object showing the related object ratings and comments.

## Multimedia Appendix 6

Result table of the BRATS 2012 challenge.

## Multimedia Appendix 7

Raw results table of the 2012 BRATS challenge.

## Multimedia Appendix 8

Top part of the details page.

## Multimedia Appendix 9

Bottom half of the details page.

## Multimedia Appendix 10

Presentation at the Open Repository Conference 2013 in Charlottetown (CA).

## Abbreviations

BRATS: Multimodal Brain Tumor Segmentation
CSV: comma separated values
CT: computed tomography
DICOM: digital imaging and communications in medicine
FMA: foundational model of anatomy
HDF5: Hierarchical Data Format
IIS: Microsoft Internet Information Service
ITK: Insight Toolkit
MICCAI: medical image computing and computer assisted intervention
MR: magnetic resonance
PACS: picture archiving and communication system
REST: representational state transfer interface
SPARQL: SPARQL protocol and RDF Query Language
SQL: Microsoft Structured Query Language
SSM: statistical shape model
VSD: Virtual Skeleton Database
VTK: Visual Toolkit
WebDAV: Web-Based Distributed Authoring and Versioning protocol
XNAT: Extensible Neuroimaging Archive Toolkit
