# Supplementary figures and images for: The Virtual Skeleton Database: An Open Access Repository for Biomedical Research and Collaboration
Source: J Med Internet Res. 2013 Nov 12;15(11):e245. doi: 10.2196/jmir.2930 (PMC3841349; doi:10.2196/jmir.2930)

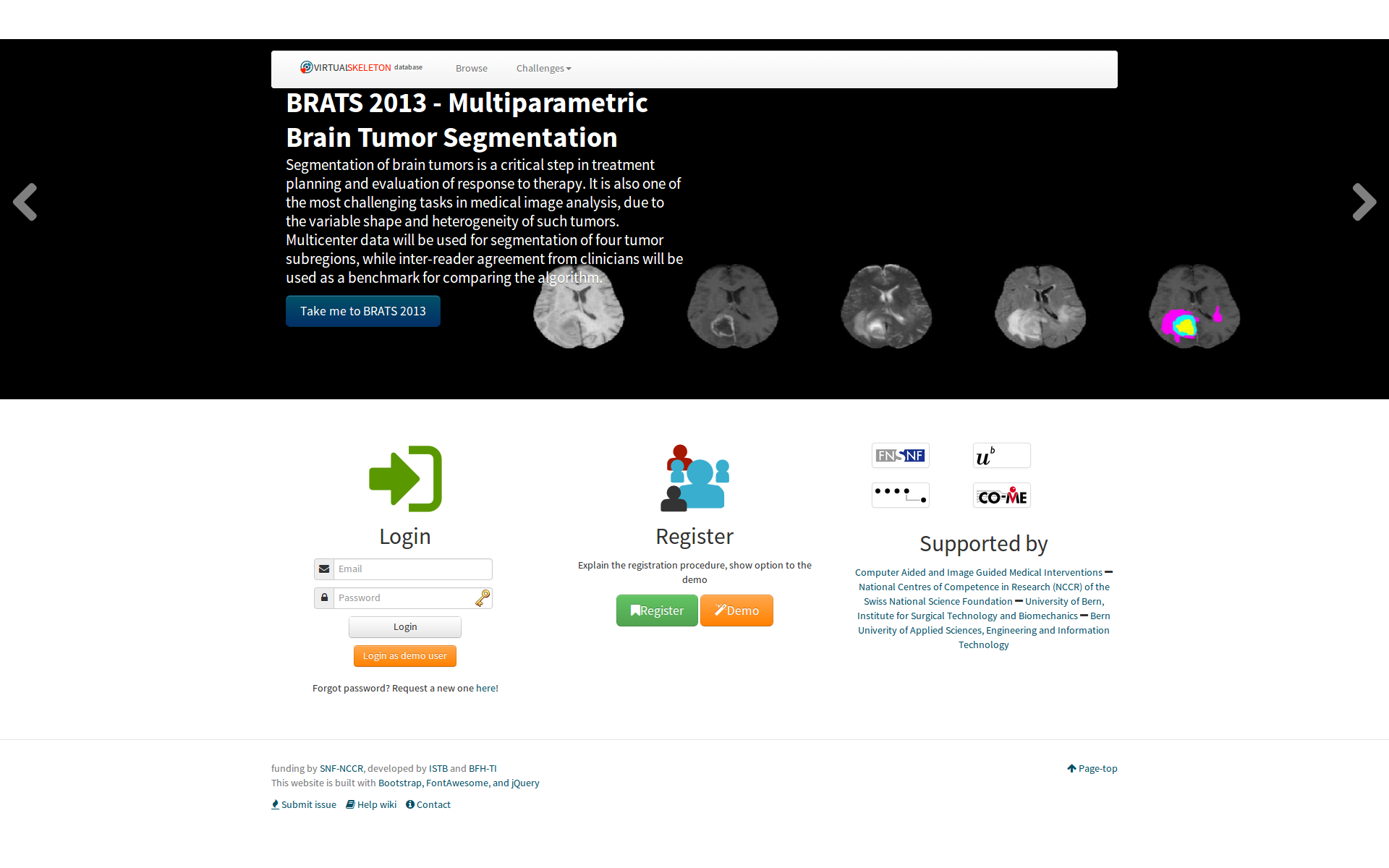

Supplement: Supplementary file 1 [file jmir_v15i11e245_app1.png]

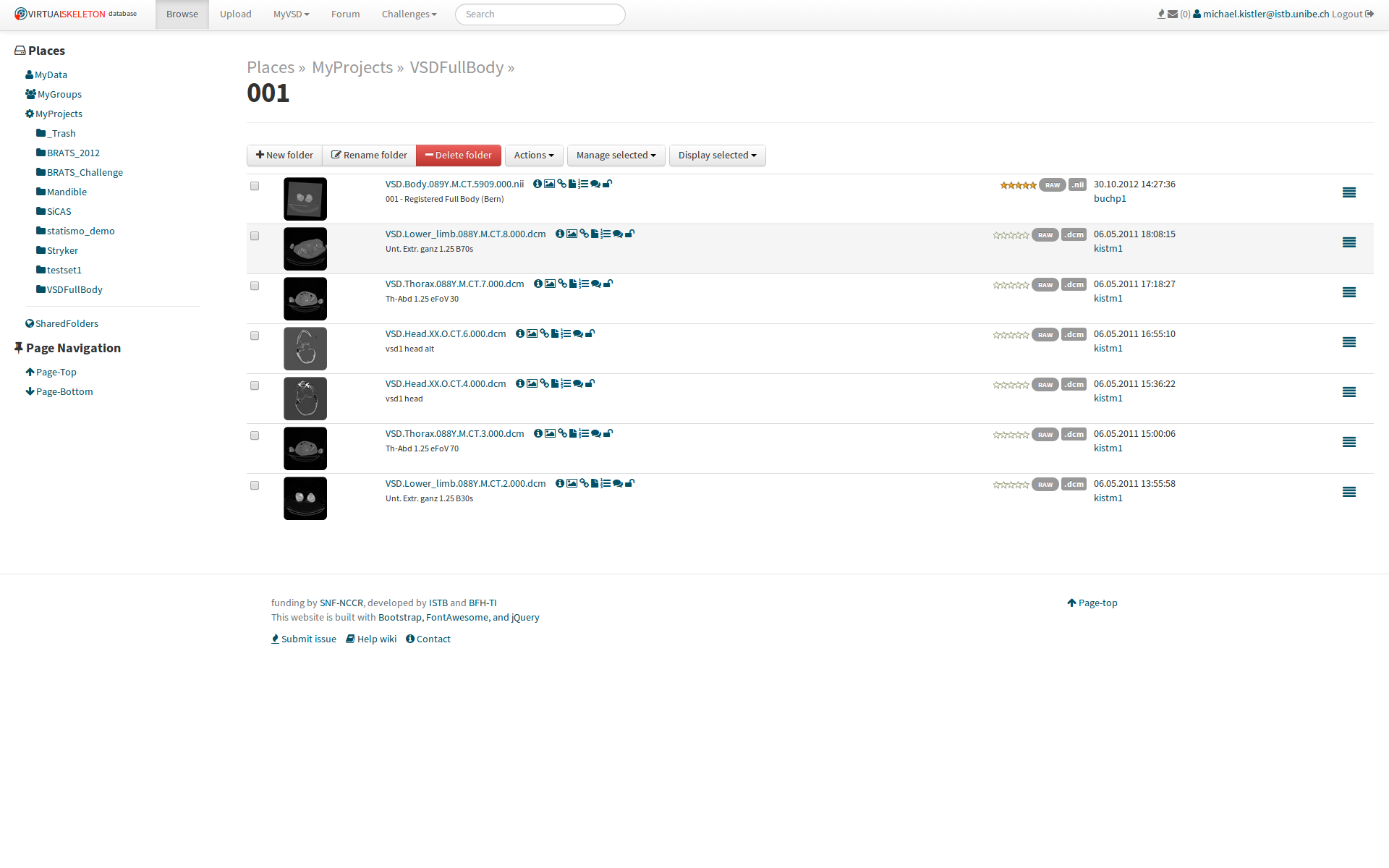

Supplement: Supplementary file 2 [file jmir_v15i11e245_app2.png]

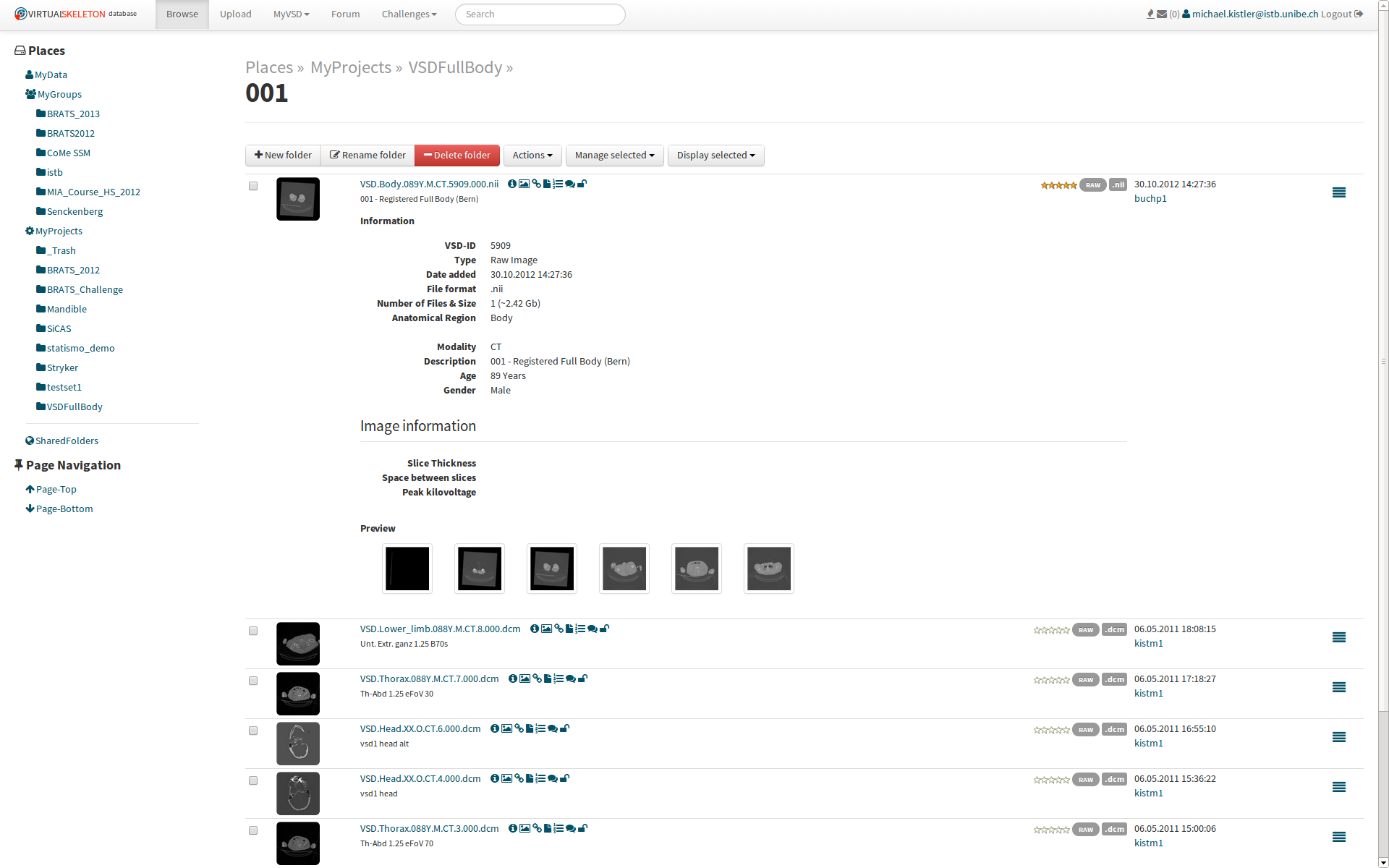

Supplement: Supplementary file 3 [file jmir_v15i11e245_app3.png]

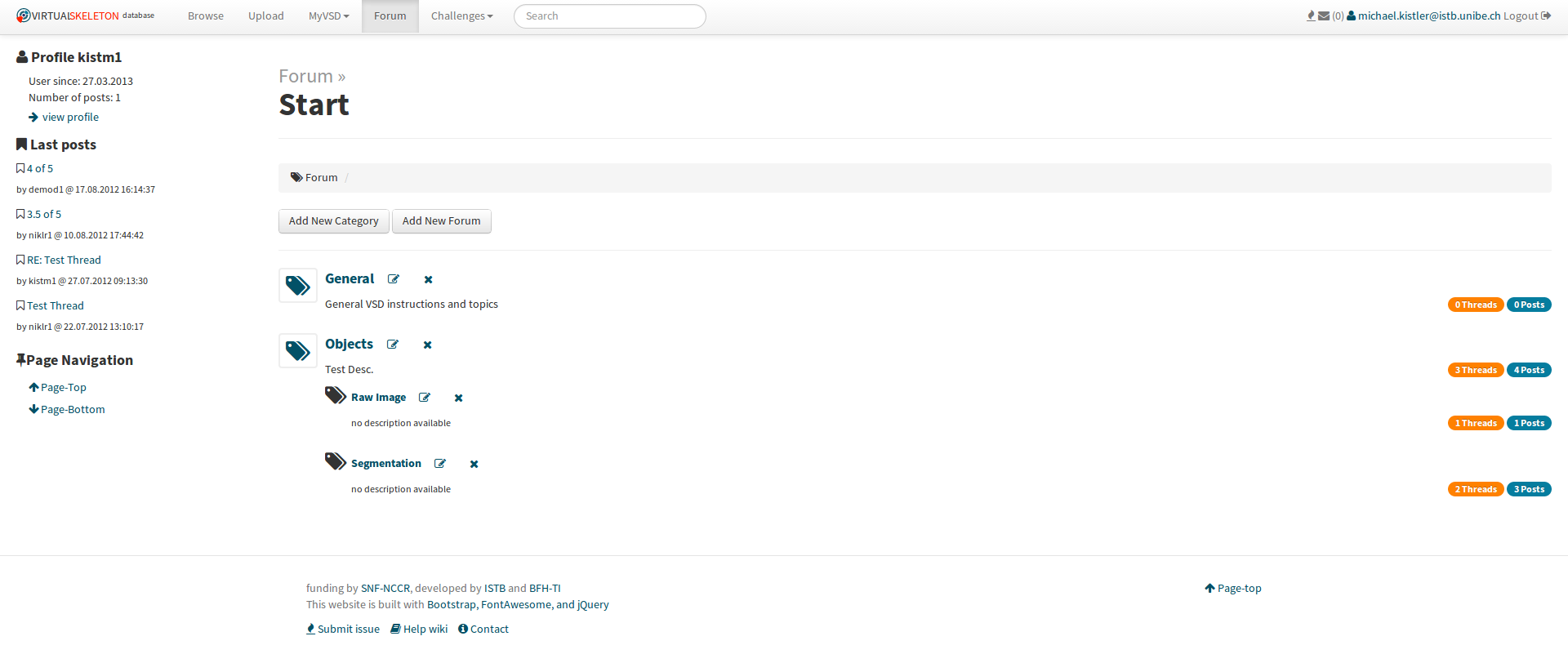

Supplement: Supplementary file 4 [file jmir_v15i11e245_app4.png]

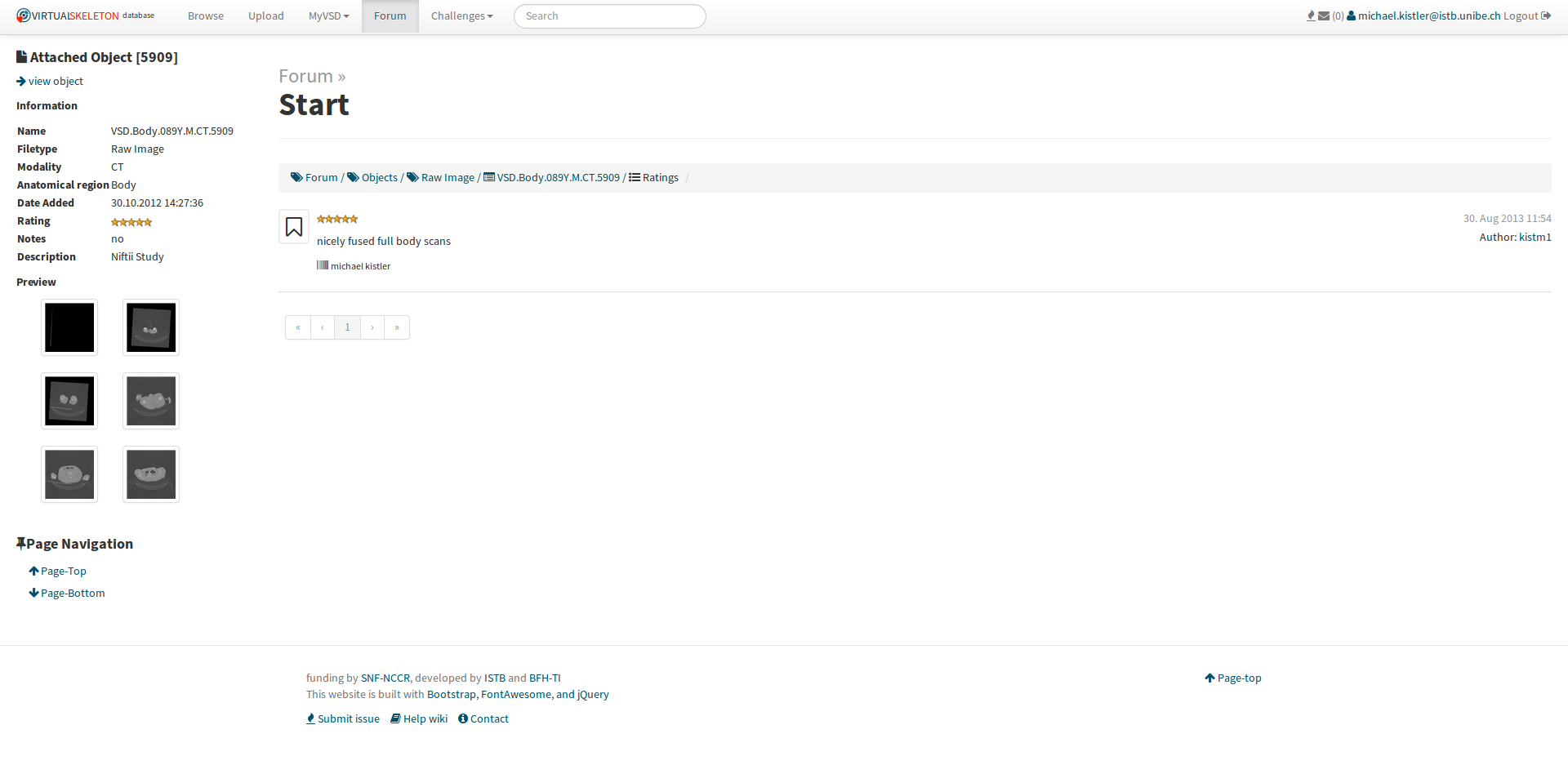

Supplement: Supplementary file 5 [file jmir_v15i11e245_app5.png]

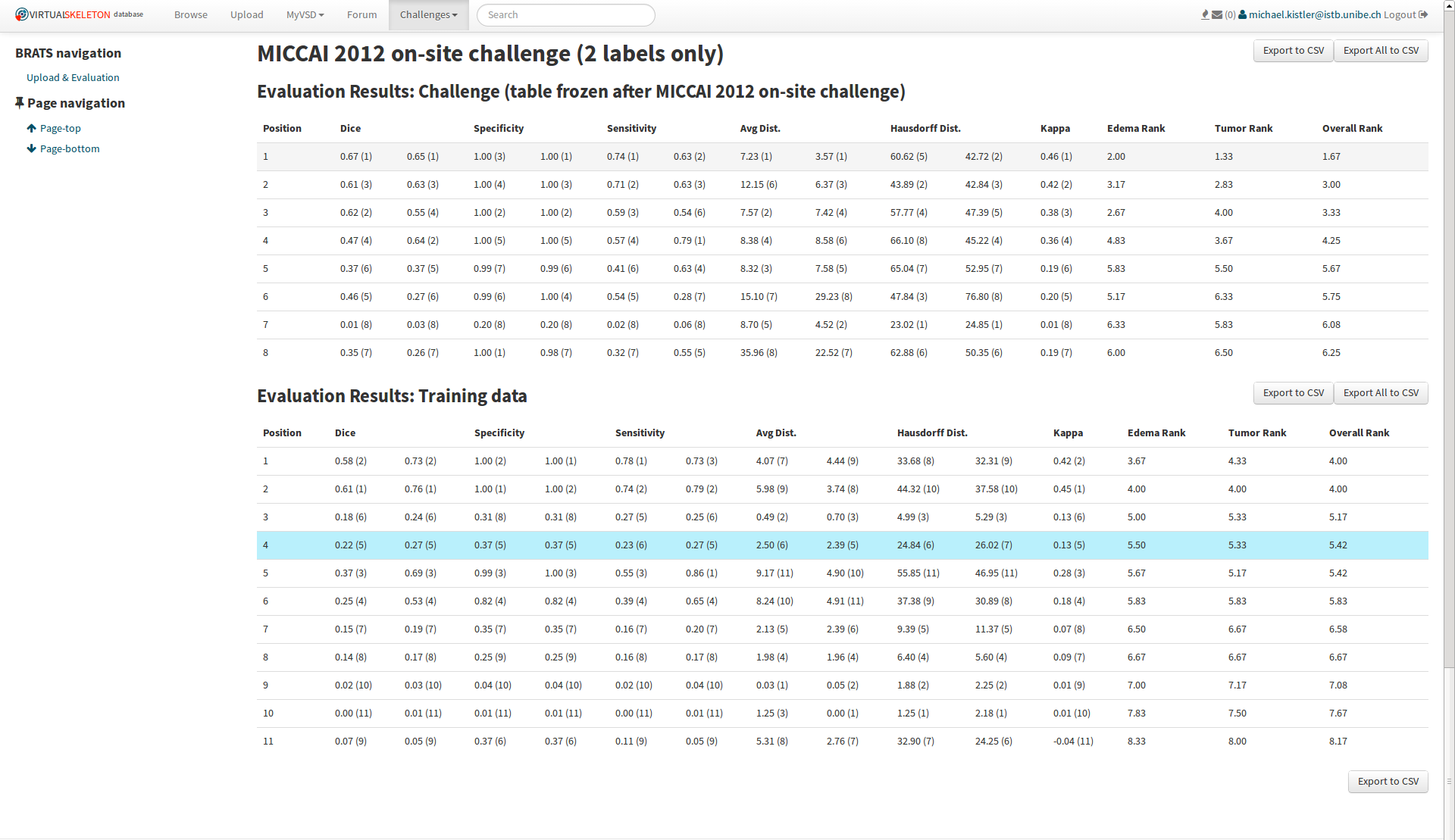

Supplement: Supplementary file 6 [file jmir_v15i11e245_app6.png]

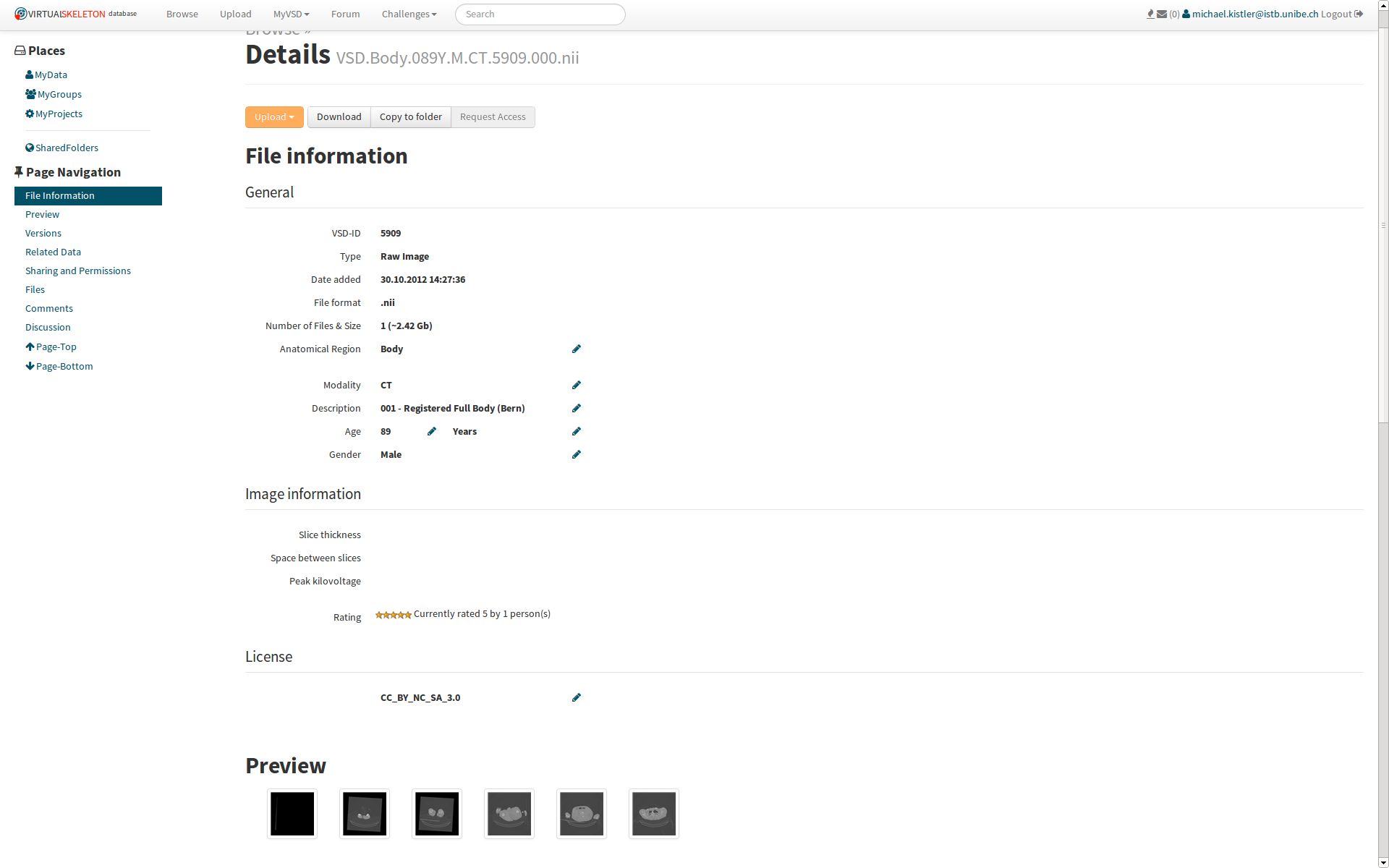

Supplement: Supplementary file 8 [file jmir_v15i11e245_app8.png]

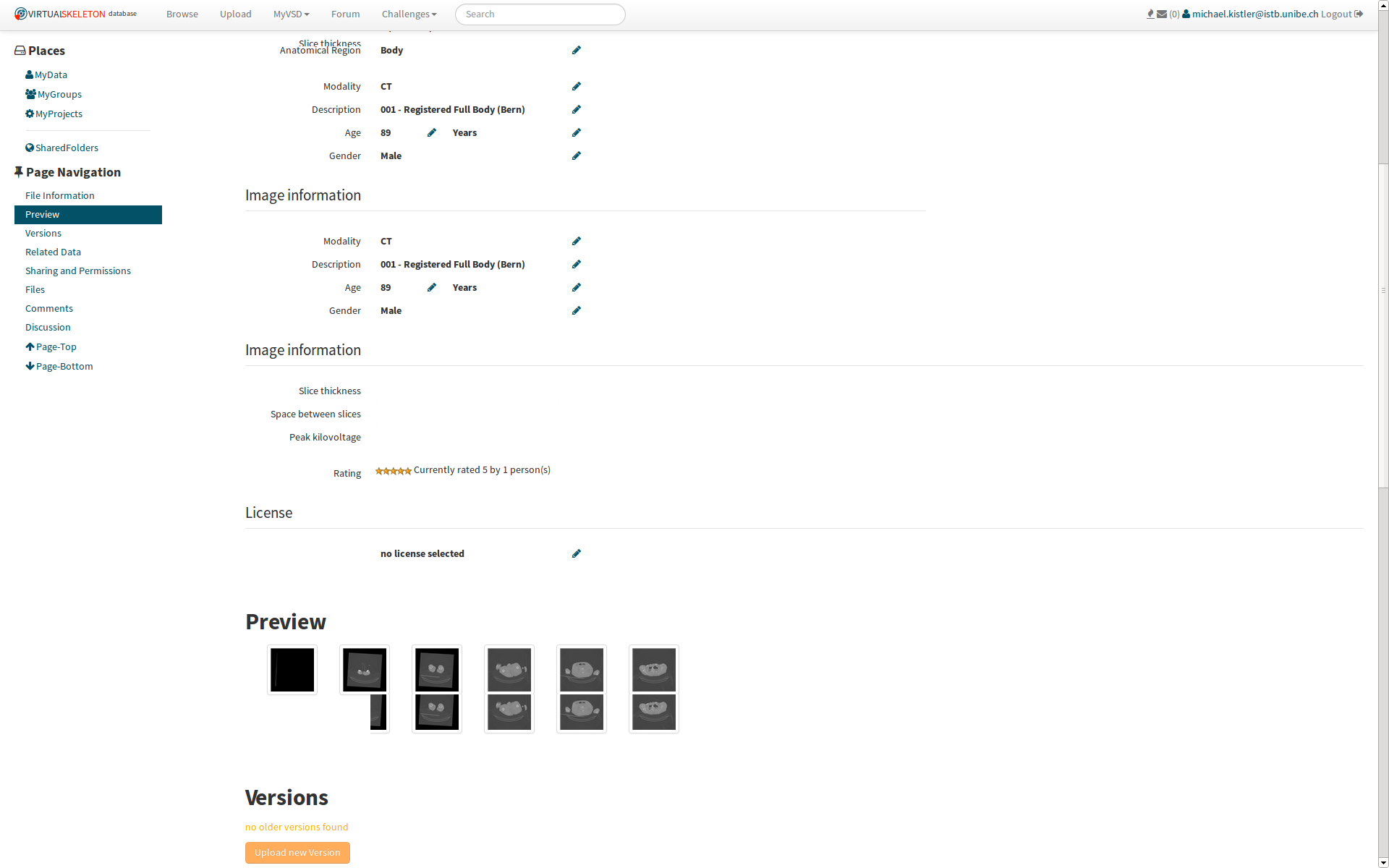

Supplement: Supplementary file 9 [file jmir_v15i11e245_app9.png]
