# Supplementary material for: The Virtual Skeleton Database: An Open Access Repository for Biomedical Research and Collaboration
Source: J Med Internet Res. 2013 Nov 12;15(11):e245. doi: 10.2196/jmir.2930 (PMC3841349; doi:10.2196/jmir.2930)

VIRTUALSKELETON database

An open access repository for biomedical research and collaboration

Michael Kistler, MSc

University of Bern, Switzerland

Institute for Surgical Technologies and Biomechanics

Michael Kistler[#], Marcel Pfahrer[+], Roman Niklaus[+], Viktor Tomas[+], Philippe Buechler[#]

[#]Institute for Surgical Technology and Biomechanics, University of Bern, Switzerland

[+]Engineering and Information Technology, Bern University of Applied Sciences, Switzerland

b UNIVERSITÄT BERN

VSD medical image analysis

- Image data
  - CT, MRI, UltraSound
- Processed data
  - Segmentation
  - Surface model
- Models
  - Statistical shape model (population analysis)
  - Biomechanical model (subject specific analysis)

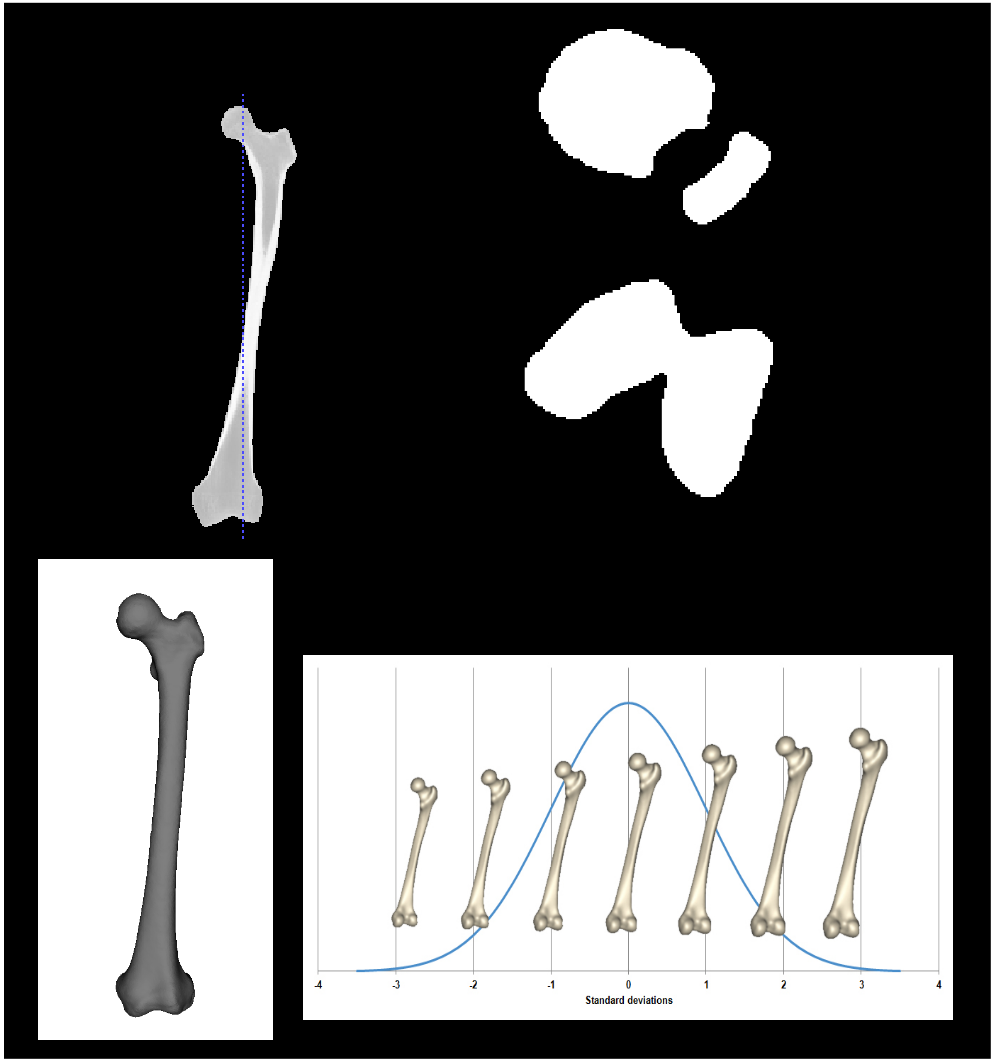

VSD Motivation

- Image management difficulties:
  - No adequate image data management systems for research
  - Redundant acquisition work, multiple copies / versions / miss-handling

- Statistical shape model: **Difficult to obtain relevant data**
  - Large amount of data required
  - Preferably good quality with meta-data
  - Redundant processing work

- Need to be able to store, track, find, exchange **images and processed data which contain anatomical structures**

Aims

- Eliminate limiting factors which prevent the fast creation
  - For testing and the application of medical image analysis methods
  - Of high quality statistical models
- Open access research repository
- Ultimately build a statistical human anatomy model

UNIVERSITÄT BERN

VSD Custom solution?

- NeuroImaging Archive Toolkit (XNAT)
  - Patient specific, PACS like storage

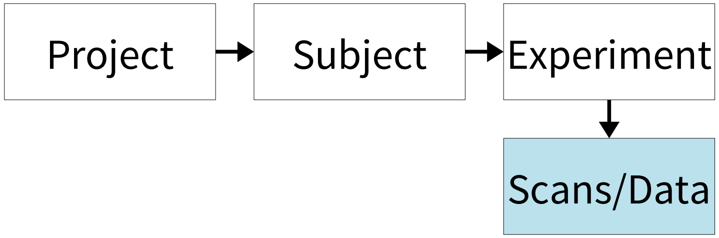

- MIDAS media archiving platform
  - Cloud storage, archiving

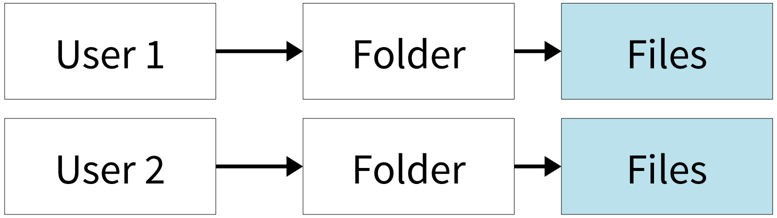

- VSD Research image repository
  - Data object centric catalog

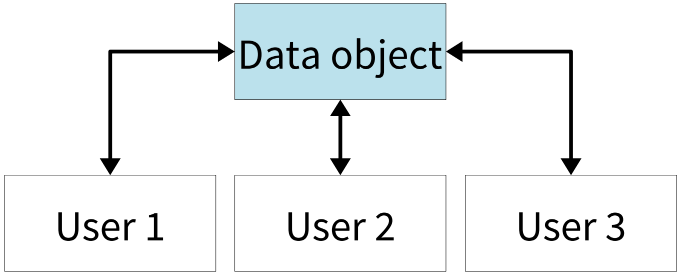

VSD Architecture

- Redundant hardware & software setup
  - MS Server 2008
  - MS IIS 7.5
  - MS SQL 2008
  - MS .NET (MVC 4, Razr)
  - SSL

- Frameworks / Libraries
  - Bootstrap, jQuery, HTML, CSS, js (Web)
  - ClearCanvas (DICOM)
  - ITK / VTK (IA)
  - Statismo, HDF5 (SSM)
  - REST (api)
  - Sphorium WebDAV
  - Fuseki (FMA ontology)

b UNIVERSITÄT BERN

VSD Uploader

EXTENSIONS	d3c dcm none	hdr, img	mha mhd, raw	nii	hdf5 h5
FORMAT	DICOM	Analyze	MetaImage	Niftii	Hierarchical Data Format
FRAMEWORK	**ClearCanvas**: File Information, Preview images, Color Count	**ITK**: File Information, Preview images, Color Count			**Statismo**: File Information, Preview images, 3d Objects
TYPE	RAW Images	Segmentation Images			Statistical Model

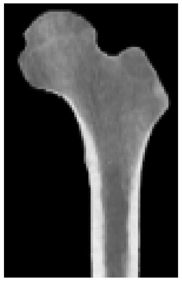

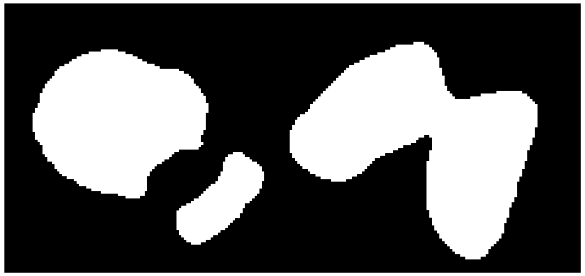

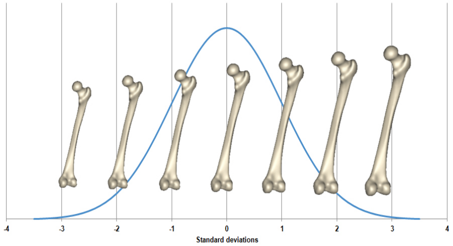

UNIVERSITÄT BERN

VSD Ontology

- Foundational Model of Anatomy
  - 75,000 anatomical classes
  - 450,000 class relations
- Retrieve anatomical tree
  - FUSEKI server and SPARQL to query the ontology and use the semantics of the ontology
- Example: Lower limb
  - Lower limb
  - Limb
  - Body
  - Anatomical structure

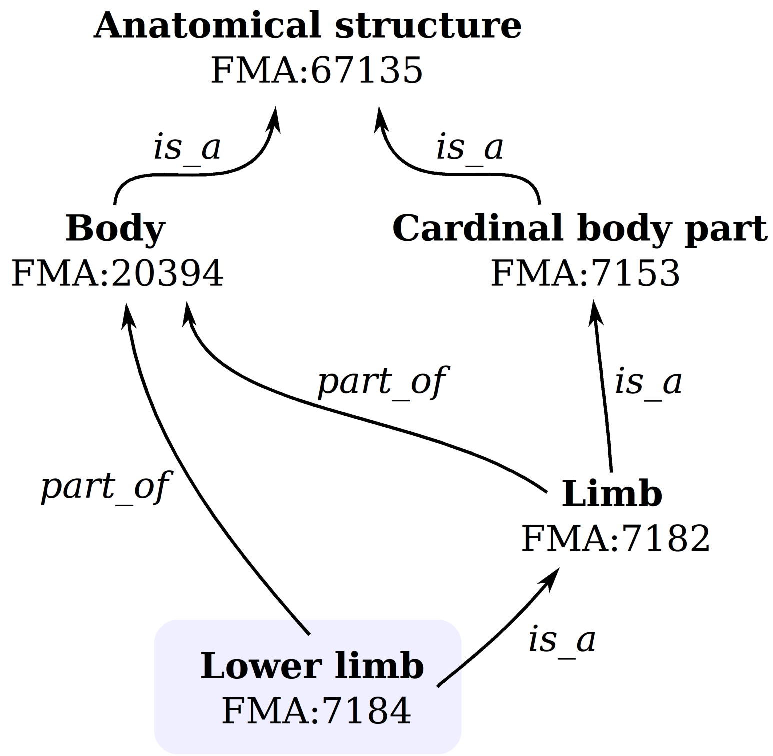

b UNIVERSITÄT BERN

VSD Ontology

- Foundational Model of Anatomy
  - 75,000 anatomical classes
  - 450,000 class relations
- Retrieve anatomical tree
  - FUSEKI server and SPARQL to query the ontology and use the semantics of the ontology
- Example: Lower limb
  - Lower limb
  - Limb
  - Body
  - Anatomical structure

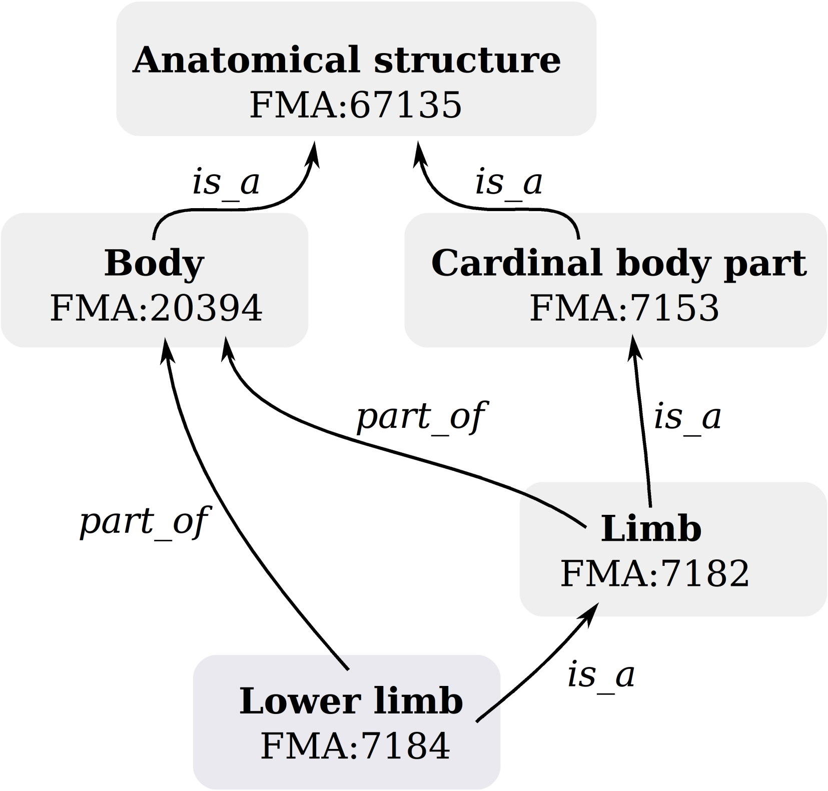

UNIVERSITÄT BERN

VSD Discussion

+

- Known software components
- Fit in curriculum
- Flexibility
- Specific requirements

–

- Longer initial development
- Less content
- Not as tested
- Code not shared with the community

> Outlook

- More content
- REST api
- Additional object types (landmarks, surface model, FE model)

> VSD

- Tailored solution for images and statistical models
- Flexible concepts - BRATS
- Lack of impressive content (Full body scanned)

b
UNIVERSITÄT
BERN

VSD Contributors

- BFH - M. Pfahrer, N. Biedermann, R. Niklaus, V. Tomas, R. Mitra
- Statismo - PhD M. Luethi
- Forensic Institute - Prof. M. Thali, PhD L. Ebert

- NCCR – CO-ME Research Network
- SiCAS Foundation

Bern University of Applied Sciences
Engineering and Information Technology

sicas
Swiss Institute for
Computer Assisted Surgery

FNSNF

UNIVERSITÄT BERN

VSD: www.virtualskeleton.ch
VSD demo: vsddemo.bfh.ch/website

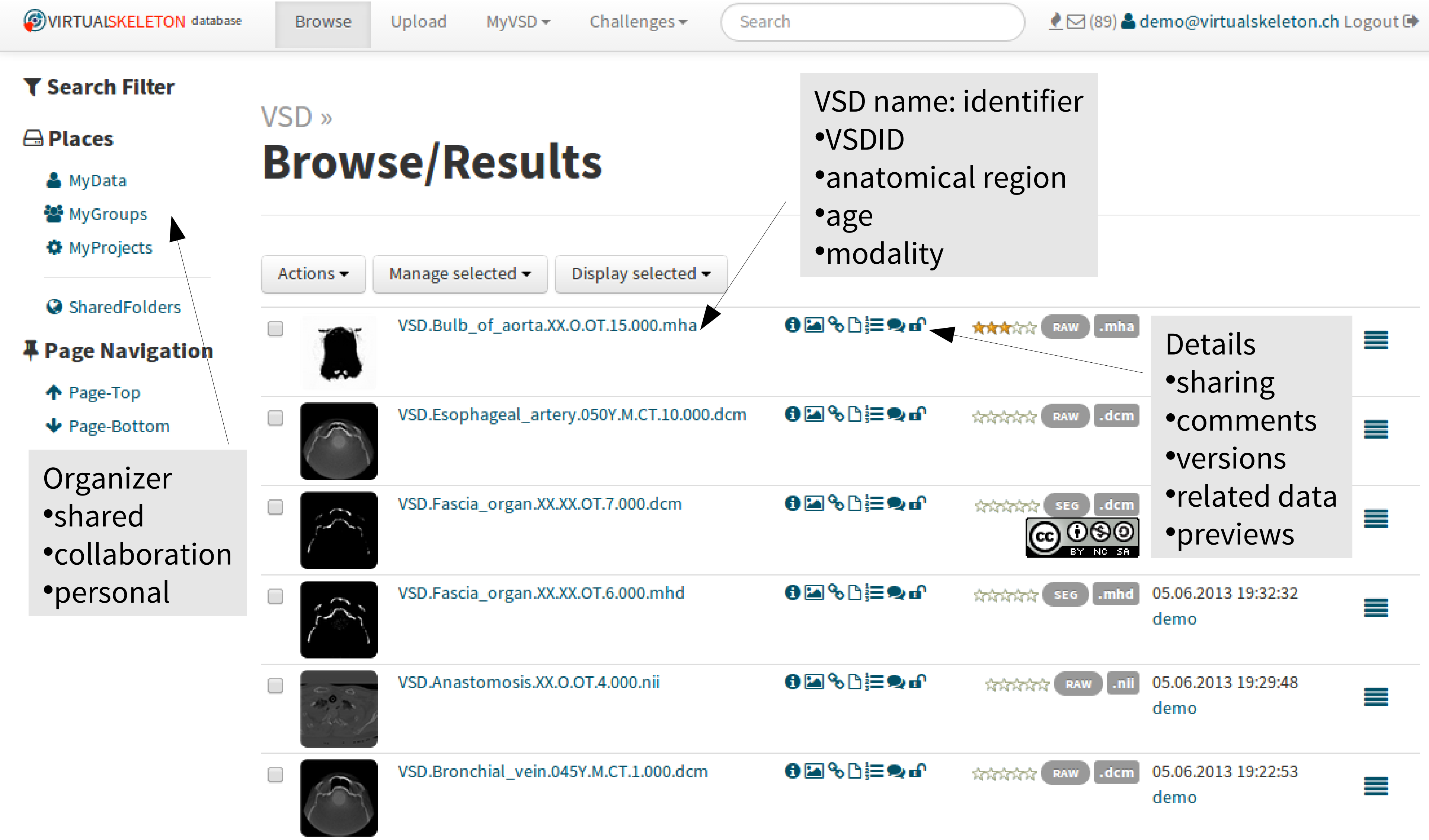

Supplement: Supplementary file 10 [file jmir_v15i11e245_app10.pdf]
